# What makes knowledge translation work in practice? Lessons from a demand-driven and locally led project in Cameroon, Jordan and Nigeria

**DOI:** 10.1186/s12961-023-01083-6

**Published:** 2023-12-04

**Authors:** Robert A. J. Borst, Rik Wehrens, Moustapha Nsangou, Dachi Arikpo, Ekpereonne Esu, Ali Al Metleq, Olivia Hobden, Martin Meremikwu, Pierre Ongolo-Zogo, Roland Bal, Maarten Olivier Kok

**Affiliations:** 1 Erasmus School of Health Policy & Management, Erasmus University Rotterdam, P.O Box 1738, 3000DR Rotterdam, the Netherlands; 2 Centre pour le Dévéloppement des Bonnes Pratiques en Santé, Yaoundé, Cameroon; 3 Cochrane Nigeria, Institute of Tropical Diseases Research and Prevention, University of Calabar Teaching Hospital, Calabar, Nigeria; 4 Department of Public Health, College of Medical Sciences, University of Calabar, Calabar, Nigeria; 5 The Higher Population Council, General Secretariat, Amman, Jordan; 6 Department of Paediatrics, University of Calabar Teaching Hospital, Calabar, Nigeria

**Keywords:** Knowledge translation, Alignment work, Uncertainty, Cameroon, Jordan, Nigeria

## Abstract

**Background:**

Over the years, the knowledge translation (KT) field has moved from promoting linearized models to embracing the importance of interaction and learning. Likewise, there is now increased attention on the transfer of KT approaches to new environments. Some scholars, however, have warned that ideas about transferability still hinge on linear thinking and doing. In the current study, we therefore sought to use a more reflexive approach to KT and to study how actors align KT approaches with their local environments.

**Methods:**

Our (auto) ethnographic study took place in a wider KT project. This project intended to combine three components: (1) co-organizing demand-driven, locally led and embedded KT cycles in Cameroon, Jordan, and Nigeria, (2) building upon established KT methods and (3) equipping and empowering local teams. We conducted 63 semi-structured interviews with key KT actors, observed 472 h of KT practices, and collected a paper trail of documents. At the same time, we also compiled project exchanges, such as project documents, plans, protocols, field notes, meeting notes and an archive of (email) correspondence between project members. We analysed all data abductively.

**Results:**

We show that there were numerous moments where the design of our project indeed enabled us to align with local practices and needs. Yet this often did not suffice, and the project design sometimes conflicted with other logics and values. By analysing these tensions, we want to show that doing KT work which acts upon different values and knowledges and is sensitive towards the different effects that it produces demands both structuring projects in a specific way and requires significant alignment work of KT actors in practice.

**Conclusions:**

We show that practising KT more reflexively relies on two important conditions. First, KT projects have to be structured with sufficient discretionary space. Second, even though the structure of a project is important, there will be continuous need for alignment work. It is important to facilitate such alignment work and to further support it. In the discussion of this paper, we therefore articulate three design principles and three sensitivities. These elements can be used to make future KT projects more reflexive and sensitive to (social) complexity.

**Supplementary Information:**

The online version contains supplementary material available at 10.1186/s12961-023-01083-6.

## Background

The idea that health policies must be informed by the best available evidence has obtained a large following in research and policy communities. Yet, how such processes might be organized, what constitutes ‘best evidence’, and the extent to which this is an advantageous endeavour, have been recurring topics of debate in the field of knowledge translation (KT) [22, 34, 37]. This field originates in the wider evidence-based movement, with a particular emphasis on studying and improving interactions between research, policy, and practice [28, 29].

With more than three decades of scholarship, it is possible to identify different generations of KT [7, 12]. The second half of this period in particular shows an abundance of approaches that unify in their strong rejection of earlier ‘linear KT’ generations. Here, linear KT is generally understood as a unidirectional research(er)-driven process of sharing ‘packaged’ knowledge using pre-defined steps [7]. The literature describes that the foremost downsides of such linear approaches are that they are too unidirectional and insufficiently demand-driven and contextualized, thereby risking having little policy impact [6, 14]. Newer KT generations, however, acknowledge that translation of knowledge requires equitable relationships, is not limited to scientists and scientific knowledge only, and that such approaches must be sufficiently contextualized [7, 21, 27, 30].

With this turn towards more reflexive KT approaches came increased attention for better understanding which KT approaches are effective, or ‘what works’ [9]. This is in contrast to earlier KT literature that is said to have followed a “throw it at the wall and see what sticks” logic ([35], p.14).

While the move towards more reflexive KT approaches is important, there have been scholars who warn that even such approaches still hinge on linear thinking and doing [44, 49]. This implicit linearization becomes clear, for instance, in how the field rarely acknowledges that KT instruments, such as rapid reviews, deliberative dialogues, and evidence syntheses, also have to be translated to become productive, that is, they must be attuned to the specific situations in which they will be applied. Neglecting the importance of such translations likely produces frustration among KT practitioners and scholars, who note that approaches that ‘work’ in one place yield more disappointing results in another (cf. [16, 50]).

There are some theories within the existing health policy and systems research literature that relate to what we call the translation of KT instruments. Generally, such literature speaks of the transferability or contextualization of KT instruments [1, 4]. They describe, for instance, that transferring a successful intervention to a new context requires a clear understanding of that intervention’s underlying mechanisms [16, 20]. Similarly, other approaches emphasize the importance of tailoring KT instruments to the contexts of their potential users [10, 24].While these literatures provide insight into ‘what works’ in terms of translating KT, they are less specific about the social, and purposive, acts of tinkering that KT actors perform when translating between their instruments and the intervention environments. It is thus not merely about ‘what works’, but more importantly, what makes it work? That is, what is the underlying work performed by KT actors to make their approaches productive? A better understanding of such underlying work is a prerequisite for practising more responsive and effective KT, yet empirical analyses into this matter remain scarce [8, 42, 43].

A literature that is particularly devoted to empirically studying underlying (mundane) work is that of science and technology studies (STS). In the STS literature, there are different approaches to studying the social, and often invisible, mundane activities that make an intervention ‘work’ in practice [18, 32, 45]. Scholarship within STS shows how the successes of an intervention commonly rely on meticulous (and often overlooked) work of (KT) actors [3, 12, 13, 33]. Such scholars argue, for instance, that merely transposing an intervention without the underlying work that it relies on results in completely different and often disappointing results.

The overall objective of this paper is to contribute to the development of a more sociological understanding of KT by analysing the translation of KT instruments through an STS-inspired theoretical lens. We thereby respond to calls for further theorization of KT and the conduct of conceptually-infused empirical studies of how KT is done in practice [5, 15, 17]. More specifically, we seek to demonstrate that KT actors in practice always work to translate their KT instruments to the contingent practices in which they take place. Such work is usually polished away in descriptions of KT interventions and valued differently, for instance, because these messy realities do not conform to stylized scientific practices. Stylized accounts – such as checklists, inventories of best practices and guidelines – may unjustly reduce the variable and uncertain nature of KT work and thus result (again) in a linearization which impairs the field from learning [47]. This brings us to our second sub-goal. By critically interrogating how we in our own project tried to translate KT tools to specific contexts in Cameroon, Jordan and Nigeria, we want to show what this demands in terms of (1) structuring and organizing KT projects and (2) the alignment work that KT actors perform.

To underline that translating KT instruments to a new environment requires processes of moulding and reconfiguring both the instrument, the environment in which that intervention takes place and the spaces in between, we will speak of processes of alignment. Our use of the term alignment is grounded in the work of STS scholar Fujimura [18]. For Fujimura, alignment (both as noun and verb) is a process of constant organizing and reorganizing between different layers of a research process (i.e. the ‘social world’, ‘laboratory’ and ‘experiment’), with the aim of making (scientific) problems ‘do-able’. Do-ability here means the extent to which relatively “well-defined tasks” (p. 258) of a research project can be conducted. To further emphasize this duality in our project, we will speak of ‘enabling alignment’ and ‘alignment work’. The former can be seen as a way of designing research projects so as to include leeway and reflexive space; for instance, by encouraging interpretive flexibility of methods by the project teams [39]. We see alignment work as a variety of purposive actions that actors within our project conducted to make the activities possible. In our analysis, we focus on the interplay between the enabling of alignments and alignment work.

Our perspective on enabling alignment and alignment work has three implications for the KT literature. First, we move away from studying how we ‘transferred’ a KT model to different countries, or how we ‘implemented’ a KT model. Instead, our perspective allows for disentangling the inherently social nature of doing KT, for instance, by showing how we tried – but not always succeeded – organizing and structuring our project in such a way that we could weave our KT approach into networks and ongoing practices in the three countries. Second, our perspective foregrounds work that is easily overlooked, or sometimes knowingly kept out of sight. We thereby position ourselves against descriptions of KT projects that neglect or obfuscate the nitty-gritty activities that enabled the project or study. This obfuscation, we argue, prevents the KT field from learning of the work that is done to make KT projects productive [47]. It is by analysing such backstage work [19] that we concretely contribute to a more sociological understanding of KT. Finally, our perspective sensitizes us to look beyond the binary of unintended and intended effects. Instead, we will focus on what ‘effects’ (in the broadest sense of the word) our project produced, and the new connections that were established in that process. These three implications taken together imply that we want to be more modest about what our KT project produced and the extent to which we were able to navigate the uncertainty that was inherent to doing this work.

## Methods

### Design and setting

To achieve the objective of this study, we used an ethnographic study design. An ethnographic study is an appropriate choice when the aim is to make sense of a complex constellation of interwoven social actions [2]. Contrary to an actual ethnography, an ethnographic study is generally less time consuming. Yet both approaches apply research methods that emphasize ‘immersion’ into a specific field, such as interviewing, (participatory) observing and analysing documents [40]. Our ethnographic study was situated within a wider research project that sought to study and improve the translation of knowledge in the field of sexual and reproductive health and rights (SRHR) in Cameroon, Jordan and Nigeria. The project, which was jointly designed in early 2017 with KT organizations in Cameroon, Nigeria and Jordan, and in collaboration with the Cochrane Africa Network and KIT Royal Tropical Institute, responded to a call for proposals by the Dutch Research Council (NWO). This call was directed at strengthening the body of scientific knowledge on what works in supporting the use of research for global development.

The designers of the KT project aimed to combine three components. First, there was an overall structure whereby the project would support local organizations in Cameroon, Nigeria and Jordan to organize a demand-driven, locally led and embedded KT cycle. Second, the project set out to build upon previously developed KT methods and principles to engage stakeholders [23, 25], establish research priorities [48], synthesize research evidence, contextualize research with local stakeholders [36, 41] and assess the uptake of research in policy and practice [26]. More information on this process and its different methods is presented in Additional file 1. Finally, the project aimed to provide the country research teams with flexibility and discretionary space. This third element was seen as most important and followed from a previous study which showed that attempts to enhance the uptake of research findings should not be planned according to tight schedules [10]. Although each country would go through the same KT cycle, the country teams were encouraged to adapt the processes to better suit the local needs, customs, and social conventions. This meant that the project should above all seek to equip, support and empower the local teams, who had to play a key role in making KT processes work.

At the start of the project, the teams from Cameroon, Jordan and Nigeria chose sexual and reproductive health and rights (SRHR) as a relevant and urgent theme in their health sectors. The KT cycles in the countries would start with establishing research priorities, followed by systematically reviewing the available evidence for proven effective interventions conducted by one team. The review’s outcomes, together with locally specific insights, studies and data, would be used to formulate country-specific evidence briefs. These evidence briefs would form the starting point of a deliberative dialogue in which participants (e.g. policymakers, health workers, youth representatives, teachers) would discuss these insights and develop possible scenarios for how knowledge about SRHR could inform policy development and improvement. Finally, the use of research results would be assessed using Contribution Mapping [26], a method to map the impact of research.

### Data collection

In this current study, we set out to analyse both how the KT project design enabled processes of alignment and what alignment work various KT actors performed. Studying this work requires a specific methodology that is sensitive to things that are not commonly noticed. In our case, we used an (auto)ethnographic methodology that involved ‘hanging out’. While hanging out has a longer track record in anthropology [38], we use it here more loosely to emphasize the relations we build with the many actors in our fieldwork. Hanging out meant immersing ourselves in their practices (cf. [51], talking, sharing stories and travelling and eating together. In doing so, we constantly paid attention to the efforts of the project members to make something ‘work’, including many moments of failure, repair and abandonment of initial plans. It is precisely such tinkering between project plans and practices that we seek to zoom in on for this current study.

Our methodology of hanging out involved different formal and less formal moments of data collection. This included 63 semi-structured interviews with key KT actors, 472 h of observed KT practices, and a paper trail of documents that we interacted with throughout the project. The interviews were guided by a topic list that included probing questions about the history and context of KT in the country, familiarity with KT instruments, KT demands and needs in the country, and reflections on our KT project in relation to their context. Most interviewees were purposively selected, whereby we aimed to include all actors that interacted with the KT project. We also used snowball sampling to select and interview actors that were more peripherally related to our project, but did have KT experience. All interviews were conducted in the key language of the interviewees (i.e. English, French or Arabic). At the same time, we also compiled project exchanges, such as project documents, plans, protocols, field notes, meeting notes and an archive of (email) correspondence between project members. More details on the data collection and the organizations that were part of our project can be read in Borst et al. [12, 13].

### Data analysis

All data were abductively analysed [46]. An abductive technique involves moving back and forth between a sensitized coding of all data using existing theory and an open coding of data that does not fit within an existing framework. It thus allows for working with existing theory, whilst not being blinded by it. The coding and categorization of our data focussed on those elements of the data that addressed: (a) design aspects of our project that provided spaces to align with local needs and capacities and (b) work that the project members performed to make the project locally possible, including adapting the KT instruments within the project to an acceptable format. We wrote detailed descriptions of the project’s different activities, identifying key moments that show how alignment was enabled by our project, what kind of alignment work was conducted in practice and to what extent such alignments produced new challenges. The different data sources and methods allowed us to triangulate our observations about what constituted forms of alignment.

## Results

Since the start of our project in September 2017, there were numerous moments where alignment between the project and local realities was necessary. It is not our intention to describe all these moments in detail here. Instead, we will zoom in on several key moments of alignment work. In our selection, we present examples that concern different layers of our research process and relate to the different methods that we applied. The examples are therefore not exhaustive but are selected because they provide the most opportunities to reflect on deviations and changes through the theoretical lens of ‘alignment work’. In the subsequent section, we will chronologically move through our project to also show how alignment work became both of elevated importance and increasingly difficult near the end of our project.

### Setting research priorities

The research priority workshop in Jordan was the first activity to be organized. As such it formed the rite of passage between our project as designed and the project as practised. Earlier on in the preparatory activities for this workshop, we learned that there was significant overlap between the workshop that we planned to conduct for our project and the activities that a partner organization (i.e. Share-Net Jordan) planned to organize as part of their own KT initiative [31]. To prevent duplication and overburdening the stakeholder network, we decided to organize a combined research priority workshop. Because the project design did not prescribe a specific format of the workshop, we could tag along with the existing initiative of Share-Net Jordan—an initiative that had already resulted in a preliminary set of research priorities that had been ratified by the Jordanian Parliament and which were thus tightly embedded in an ongoing policy impetus. In this case, this alignment work involved combining different activities, agendas and funding:

“We combined all the project funding from Share-Net International and the project funding by NWO-WOTRO. Each team could then present the problems. Therefore, we were able to invite all [stakeholders], around 90 persons, to attend this meeting. Now they know everything about the project” (interview with Jordanian KTP actor).

What this example shows is that KT processes never happen in isolation, but that there are often numerous other interacting initiatives by national and international organizations. This ‘layeredness’ of KT interventions is often overlooked, and in our case, this could have resulted in organizing a workshop that was detached from an existing agenda to improve SRHR in Jordan. Instead, and through the space that our project structure provided, we could perform work to align our project’s activity with an ongoing KT process of a different organization.

Similar to the situation in Jordan, the Cameroonian and Nigerian research conducted research priority workshops that worked for their situations. In project meetings with the Cameroonian team, they describe that a preliminary contact with the Ministry of Health showed that they wanted to revive a dormant, yet existing, platform for SRHR but had insufficient capacities for this. In their reports of this contact, the team writes that this is an “opportunity to anchor” the new KT cycle in an existing infrastructure. Therefore, their alignment work was directed at presenting themselves as a solution to the ministry’s capacity problem. The obvious benefit to the team’s KT cycle was that the association with the ministry would further legitimize their approach. The team eventually postponed the research priority workshop for 4 months and used part of their budget to organize an extensive consultative process with the related ministries. This shows that alignment work may involve slowing down, in this case clearly with the anticipation that attaching the KT process to the dormant platform would enable the team to build on existing networks and (ministerial) infrastructures.

The Nigerian team, in turn, learned through preparatory interviews with envisioned workshop participants that a stratified approach made more sense. In the interviews, several stakeholders explained that one single research priority workshop would nullify the distinctions in needs and capacities between two of the three administrative levels of Nigerian government. Or as one of the interviewees said:

“When the activity is conducted as you said, it does not apply to our setting” (interview with Nigerian subnational policymaker).

Their suggestion therefore was to organize one dialogue at the federal level and a separate workshop at state level. During a project meeting shortly after, the Nigerian team decided to follow the stakeholders’ suggestions and to stratify their research priority setting. Through this alignment work, the team created buy-in from the stakeholders, but more importantly: they aimed to prevent producing a list of research priorities that would be recognized neither by the national nor subnational level.

These descriptions show different types of alignment work. Both in Jordan and Cameroon, the teams could attach our plan for a research priority workshop to ongoing activities of other organizations, including a dormant ministerial platform. In Nigeria, alignment work involved stratifying the workshop across the two administrative levels. This also brought about new challenges. The approach in Nigeria, for instance, amplified a dispute about what was considered appropriate evidence. For the stakeholders at the subnational workshop, it was essential that the Nigerian team mobilized evidence produced by local organizations. The national stakeholders, however, wanted the team to use evidence from international scientific literature. This led to a doubling of synthesis work for the team, and demanded further alignment work between their role as a facilitator of evidence syntheses (i.e. Cochrane Nigeria) and a subnational-based university research centre. This alignment work significantly strained the available project budget.

Our depictions of alignment work in the organization of research priority workshops shows two important considerations. In the first place, we have shown that the ‘interpretive flexibility’ [39] of KT methods built into our project design enabled the research teams to attune to ongoing initiatives in their countries. This did, however, require specific capacities of the teams which not all researchers may commonly possess, for instance, to stay sensitive to local needs and practices whilst also being able to account for progress on specific project goals. The second consideration concerns the uncertainty that comes with alignment work. While such work appears feasible in the short term, long(er)-term consequences are difficult to foresee and anticipate. This became visible in the Nigerian example, where the epistemic fundament of the entire KT approach came under considerable scrutiny. In addition, uncertainty also affects budget plans and thus necessitates that each project phase has sufficient budgetary space to do alignment work.

### Systematic review

Shortly after the Nigerian team began their systematic reviewing process, they began expressing concerns regarding the feasibility of conducting a Cochrane systematic review given the project’s timelines. The key research priority that was identified in our project had been the topic of a recent Cochrane systematic review. The Nigerian team thus explained that it would be “senseless” and a duplication of effort to conduct a Cochrane review, and the authors of the published review “would probably object to ours” (observations of project meeting). Alternatively, the Nigerian and Cameroonian teams suggested carrying out an ‘overview’, a review of systematic reviews, focussing not only on “what works, but equally on why it works and how it works” (Cameroonian KTP director). This choice produced two important challenges. First, an overview according to Cochrane methodology still required an extensive registration and editorial process which did not necessarily align with the project’s timeline. Once registered, we would no longer be able to alter the scope of the overview, as this would be seen as “bias” (meeting notes). Yet, given that our aim was to specifically align the KT process to local dynamics, it was likely that the overview would need slight alterations as well. Second, the overview required a clear demarcation of topics. In an attempt to nonetheless do justice to all research priorities and to fulfil demands of stakeholders in the three countries, the project teams decided, as described in the excerpt below, to conduct a rapid ‘scoping search’.

“In order to meet the needs of the stakeholders, we discuss the possibility to do a scoping search: basically, identifying the evidence gap with regard to sexual and reproductive health of [internally displaced persons]. The scoping search takes less time and it is therefore decided among all team members that it should be possible to have the scoping search ready by the end of August” (minutes of project meeting).

The process of organizing the systematic review shows an important reflection in terms of alignment work. Our research project highlights that highly structured review methodologies are not necessarily well equipped for aligning with the needs and practices of potential users of the knowledge from that review. Most saliently, in its attempt to reduce ‘bias’ and increase the alleged replicability of research, such methodology impairs the space that is available for alignment work. The solution in this project was to opt for less structured methodology, which also has consequences for the extent to which such findings can be published in the scientific literature and the legitimacy that stakeholders in the field subsequently bestow upon our results.

### Conducting deliberative dialogues

The organization of deliberative dialogues clearly marked a new phase in our project. The project was about 1 year in, but by now there were also elections in Cameroon and Nigeria. Additionally, the overall project group was less experienced in organizing deliberative dialogues. These two elements thus created new kinds of uncertainty. This uncertainty provided challenges and opportunities at the same time, but also required different forms of alignment work.

The first dialogue was organized in August 2018 by the Cameroonian team – a team with extensive experience organizing deliberative dialogues. Over time, the team had meticulously tweaked the deliberative dialogue method as to fit their context better – for instance, by moving away from the notion that such dialogues must not establish consensus among stakeholders (see also Additional file 1). Given that the project provided quite some leeway as to how the dialogues should be organized, the teams could organize and conduct the dialogues as they seemed fit. As explained earlier on, the Cameroonian team noticed they could align their KT process to the revival of a Ministerial SRHR platform. Therefore, they decided to focus the deliberative dialogue on “strategic courses of action” (research priority report) specifically designed for that platform.

Contrary to what was anticipated, the dialogue mainly revolved around introductions and attempts at defining a shared problem definition. While the attendees of the dialogue (partly) knew each other, this proved to be the first time that they attempted to arrive at a “shared understanding” (dialogue transcript) of SRHR issues among young populations. As becomes clear in the transcription of the dialogue, the attendees found it of crucial importance to identify the “eligible age group” (dialogue transcript). By the end of the dialogue, the participants agreed on an age group, and this was seen as a “substantial achievement”. One of the team members noted that the dialogue had not addressed most of the priorities and policy options, and thus they organized a follow-up session. Now that they had resolved issues around terminology, they could – specifically for the defined target group – present interventions for which the Ministerial platform would be responsible. This required alignment work in terms of funding, as described below.

“Since this was originally not in the budget, we need to be a bit creative” (e-mail correspondence).

The Cameroonian team hosted a second deliberative dialogue 9 months later, and despite the delay due to presidential elections, there was still sufficient momentum to discuss concrete interventions that the Ministerial platform could undertake.

In contrast to the process in Cameroon, the organization of the deliberative dialogues in Jordan and Nigeria presented more challenges. Saliently, it was the same flexibility which had made that the Cameroonian approach productive that now presented itself as an obstacle in the other two countries. A combination of logistical issues, schedule conflicts and concerns regarding “country differences” (project call) thwarted that collaboration.

“[The professors] had a discussion about facilitating the meeting. [The professor from Cameroon] was willing to do this. Unfortunately, it is now too dangerous by road, and too costly via air” (email correspondence).

Eventually, the Nigerian team pragmatically organized the dialogue themselves, with emphasis on how they – in their position as Cochrane Nigeria – could work together with both federal and state-level policymakers.

For the Jordanian deliberative dialogue, the team proposed attracting an experienced facilitator from a neighbouring country; a plan which was abandoned after some weeks, given that this would cost almost US$ 100 000 (more than 60% of the entire project budget). To proceed with the project, the Jordanian team suggested jointly organizing the dialogue with the Dutch team (project call). This team would draft a programme for the dialogue and produce an evidence brief using local evidence, whereas the Jordanian team would be responsible for arranging the setting and ministerial permission, inviting the participants and facilitating the meeting.

In the final ‘dry run’ of the dialogue, however, the Jordanian team noticed that the Dutch team had planned a full day programme, which they said would not work in terms of timing, as can be read in the quote below.

“You need to shorten the programme, a lot, ya’ni [Arabic for ‘you know’]. Because everything needs to end before lunch. Especially now during Ramadan, no one will come after lunch, and you have planned the most important part of the dialogue there” (preparatory interview with policy advisor, Jordan).

The challenges presented above show that the organization of deliberative dialogues required different types of alignment work per country: organizing a follow-up dialogue to relate to ongoing political developments (Cameroon), establishing new connections with the needs of (sub)national policymakers (Nigeria) and enhancing feasibility by connecting to cultural norms (Jordan).

### Mapping our contributions

The first group to start with the Contribution Mapping process was the team in Cameroon. This was the first time that the group would use the method and we jointly decided that a researcher from the Dutch team (with previous experience using the method) would team up with a researcher from Cameroon. After translating the interview guide into French, they conducted pilot interviews to check guides’ appropriateness for the context. The main issue they experienced was that both the interviewees and the Cameroonian researcher were not accustomed to having semi-structured interviews that easily took 1–1.5 h. In addition, the researcher from the Dutch team was not proficient in French and had no knowledge of the Cameroonian context and customs.

“N: *clicks tongue* It was too long. Ce n’est pas simple. Huh. It is difficult! *laughing*

B: Euh, what, you mean the [interview] guide? Or?

N: Yes. These are big men. They will not have hours. It is not normal”

(conversation after interview, 13 November 2018, Yaoundé)

After several interviews, the Cameroonian researcher described that he, and several of the policymakers with him, was more familiar with structured interviews, while Contribution Mapping assumes an open approach and takes significant time. Most of the alignment work at this stage was thus directed at adapting the interview guides to fit better with the skills of the researcher and research climate in Cameroon, but at the same time the Cameroonian researcher tried to find a compromise between the unstructured nature of Contribution Mapping and his own expertise. Despite this alignment work, the use of Contribution Mapping in Cameroon remained challenging. This also had to do with the fact that the method assumes that research is organized in project-like entities, whereas a substantial part of the SRHR research that we identified was either self-funded (e.g. PhD research using personal savings) or part of structural monitoring and evaluation activities of non-governmental organizations (NGOs).

The Contribution Mapping processes in Jordan and Nigeria were due to start shortly after the process in Cameroon. However, logistical concerns, the difficulty in making Contribution Mapping more context sensitive and other priorities and diverging perspectives of the various project members significantly delayed the start. Once these issues were overcome, the project reached its end and could no longer be extended.

Our examples of alignment work bring to light several challenges. Foremost, our attempts at organizing Contribution Mapping in a decentralized way shows that this method assumes specific capacities, both of its users and the environment in which the method is used. Furthermore, our use of Contribution Mapping illuminated implicit epistemic normativities in our project design: project members had diverging ideas as to what could be considered valid scientific research, what role research knowledge may play in improving SRHR policy and practice and how research impact might be understood and assessed. Finally, activities such as Contribution Mapping assume a longer follow-up period and thus often traverse the formal project timeline. However, once the project has ended it becomes impossible to pay invoices or have project costs reimbursed.

## Discussion

With this paper we respond to calls for further theorization of KT and the conduct of conceptually infused empirical studies of how KT is done in practice [5, 15, 17]. In particular, we do so by analysing how we tried to organize three KT processes in Cameroon, Jordan and Nigeria. We show that the extent to which our approach ‘worked’ depended on meticulous efforts to align with the environments in which we sought to intervene. This alignment work situates (in)between different layers of project and practice and is commonly overlooked. The aim of such alignment work is to reconfigure these layers until they provide a productive fit (i.e. they temporarily align).

In this discussion we will explicate what our approach of ‘alignment work’ has to offer the KT field, especially in relation to engaging with (un)certainty. We tease out design principles and sensitivities that offer a different way of accounting for both ‘what works’ and what is needed to make something work.

Our project explicitly aimed to enable alignment through its design. We did this, for instance, by offering the teams space to interpret and adapt the project’s KT instruments to forms that fit their environments. The analysis of our project shows that the teams subsequently performed alignment work at different places, in numerous forms and at varying levels of complexity. Most of the alignment work was relatively pragmatic in nature: to prevent duplication, to deal with absence of sufficient funding or to work around conflicts and standstills, the KT teams restructured their activities and re-aligned them with ongoing local initiatives, sometimes of organizations working on similar topics. At other times, alignment involved carefully working with time, timing and momentum. Finally, alignment work was sometimes epistemic in nature and involved producing more ‘localized’ or situated knowledge.

### Implications of a more sociological KT approach

Our perspective on the enabling of alignment and the acts of alignment work has several implications for KT practice and research. In using this perspective, we noticed that uncertainty played a different role compared with common ‘new’ KT approaches. Such approaches often strive to reduce uncertainty as much as possible, for instance, by relying on protocols and checklists to standardize KT work. This reduction, however, takes away the possibility to align with local developments and needs. In enabling alignment, we realized that the ‘effects’ of our project would be difficult to foresee and thus also challenging to account for towards our research funder. This eventually led to numerous meetings and phone calls with the funder’s programme manager in which we tried to explain the many ‘deviations’ from our proposal, which in itself can be seen as a type of alignment work. Noting the importance of aligning with local developments and needs, we deem it important to organize and practice KT in a different way. We have therefore articulated several ‘design principles’ [10] and sensitivities to serve as guidance within the inherently uncertain KT processes. The former includes aspects that may be considered when designing a KT project, and the latter concerns elements which can be reflected upon during a KT project.

### Design principles

#### Plan alignment work

Doing alignment work is expensive. Our analysis shows that there were several moments in which additional activities had to be organized, which we did not anticipate yet significantly strained our budget. Earlier research shows that this may require creating overheads [12, 13]. The literature also emphasizes that KT projects should start with a strong basis, for example, by engaging stakeholders from the onset [10, 11]. While this seems a useful suggestion, it often results in insufficient budget and time near the end of a project. We therefore propose planning alignment with an explicit end-focus: devote unearmarked time and resources to the final period of a project.

#### Inscribe interpretive flexibility

The instruments that we used in our project were not always accompanied by clear protocols or guidelines. As shown in our analysis, this sometimes required producing them on the go. More often, however, there were moments in which these quite vaguely defined instruments offered just enough guidance so as to adhere to a (formal) KT strategy, and sufficient possibility to interpret the instrument in accordance with local circumstances.

#### Create space for alignment work

Aligning often means doing something that was not foreseen. Most projects are organized in work packages, with clear deliverables and deadlines. This may create tensions between the ability to change directions, and the requirement to abide to a project logic. We therefore suggest creating spaces for alignment at different places and moments within a project (proposal), for instance, by describing that an activity depends on priorities that are defined in the course of the project.

### Sensitivities

#### Epistemic sensitivity

Actors within a KT project may have different (normative) understandings and convictions of what ‘good research’ and ‘research impact’ entails. This may create tensions between accommodating these different understandings and the projects’ productivity. We therefore deem it important to constantly (re)define a shared understanding and normative agenda, for example, that this is a KT project that uses a more constructivist understanding of scientific research.

#### Communicative sensitivity

There are numerous earlier studies that stress the importance of having regular face-to-face interactions within a project team (cf. [16]). However, in practice, convenience is often a strong attractor and in-person and long-term engagements suffer. However, we have seen that KT projects such as this benefit a lot from spending time and hanging out together, in person, on a regular basis. We therefore suggest being sensitive to the social cohesion and communication within the project team, as this reflects on the KT work.

#### Reflexive sensitivity

Projects work through deadlines and commonly require fast-paced working, with well-delineated time frames. In doing KT work, this prevents establishing new relations beyond the project and considering whether there may be other initiatives with which the project can be aligned. We therefore suggest regularly slowing down and zooming out.

### Reflection on our analysis

We see two potential limitations to our sociological ‘alignment work’ approach. First, we observed alignment work by hanging out in the everyday practices of the different research teams. This meant that we had to make decisions as to where we drew the boundary between ‘work’ and other types of (less purposive) actions. In our analysis, this was a ‘line in the sand’: the boundary between work and non-work was constantly redrawn. Star and Strauss [45], p. 14, describe that “[w]hat will count as work does not depend a priori on any set of indicators, but rather on the definition of the situation”. It is therefore important to note that what we see as work may not count as work elsewhere. Second, and related to the first issue, is how we distinguished between those parts of our observations that we saw as alignment work and the parts we identified as other types of work. We realize that our ‘examples’ of alignment work can easily be captured in other terms, both conceptually and in the more commonsensical understanding that this is simply how research operates. What we think makes alignment work distinctive is that – following Fujimura [18] – this describes coordinating activities meant to make a research process ‘do-able’. That means that alignment work necessarily concerns work (in)between research layers, in our case that of an academic environment, research project and concrete improvement practices in their local environments.

## Conclusion

Our study shows that practising KT more reflexively works on (at least) two important conditions. First, KT projects have to be structured with sufficient discretionary space. Such spaces can be used to align with local priorities and to move along with the tides of the relevant stakeholder communities. Second, even though the structure of a project is important, there will be continuous need for alignment work. It is important to facilitate such alignment work and to further support it. We have therefore in the discussion of this paper articulated three design principles and three sensitivities. These elements can be used to make future KT projects more reflexive and theory driven.

### Supplementary Information

**Additional file 1**. Overview of used knowledge translation tools and their key principles. The file shows the knowledge translation tools that were used in the project and includes a description of their key principles and references to appropriate source literature.

## Data Availability

The anonymized data that were used and analysed during the current study are available from the corresponding author on reasonable request.

## Abbreviations

KT: Knowledge translation
KTP: Knowledge translation platform
NGO: Non-governmental organization
NWO: Dutch Research Council
SRHR: Sexual and reproductive health and rights
STS: Science and technology studies
