# Supplementary material for: What makes knowledge translation work in practice? Lessons from a demand-driven and locally led project in Cameroon, Jordan and Nigeria
Source: Health Res Policy Syst. 2023 Dec 4;21:127. doi: 10.1186/s12961-023-01083-6 (PMC10694879; doi:10.1186/s12961-023-01083-6)
Supplement: Supplementary file 1 — Additional file 1. Overview of used knowledge translation tools and their key principles. The file shows the knowledge translation tools that were used in the project and includes a description of their key principles and references to appropriate source literature. [file 12961_2023_1083_MOESM1_ESM.docx]

**Additional file 1: Overview of used knowledge translation tools and their key principles**

Supplementary file to manuscript with title ‘What makes knowledge translation work in practice? Lessons from a demand-driven and locally-led project in Cameroon, Jordan, and Nigeria’ by Borst et al.

Table 1 shows the knowledge translation tools that were used in the project. The second column presents key principles of the tools, and the final column suggests further readings.

**Table S1. Overview of knowledge translation tools and their key principles**

| **Knowledge translation tool** | **Key principles** | **Further reading** |
| --- | --- | --- |
| Stakeholder engagement | - Actors are conceptualised (e.g. potential users) and identified as having a ‘stake’ in the knowledge translation process - These stakeholders are invited to join the knowledge translation process - These potential users become part of one or different components of the process - Throughout the process, different strategies are used to incorporate knowledge, demands, and wishes from the stakeholders | Boaz et al., 2018  Borst et al., 2019  Oliver et al., 2019 |
| Research priority workshop | - Stakeholders are invited to join one or multiple workshops - Researchers, practitioners, and/or policymakers present wider theme of action - Stakeholders are asked to articulate areas where more, or different research is needed - In different rounds, these areas are categorised and organised - The final areas are ranked in terms of priority | Pratt, 2019  Viergever et al., 2010 |
| Systematic review | - Synthesises scientific evidence on a demarcated topic of research - Uses a hierarchy of scientific evidence - Unit of analysis is primary studies - Applies systematic methods - Appraises quality of evidence and risk of bias - Summarises key outcomes | Armstrong et al., 2011  Petticrew & Roberts, 2006 |
| Overview | - Synthesises outcomes of systematic reviews on a demarcated topic of research - Unit of analysis is systematic reviews - Applies systematic methods - Appraises quality of reviews - Summarises key review outcomes - May include re-analysis of data and postulation of new research questions | Smith et al., 2011 |
| Scoping search | - Identifies and analyses gaps in knowledge - Clarifies concepts - Unit of analysis is primary studies - Applies systematic methods, but no critical appraisal | Levac et al., 2010 |
| Evidence brief | - Short document in non-academic language - Addresses key priority as identified earlier - Presents sufficient context of the issue - Mobilises evidence from different (inter)national studies - Highlights policy options, their considerations, and costs - Offers implementation strategies and techniques | Lavis et al., 2009  Moat et al., 2014 |
| Deliberative dialogue | - Informed by an evidence-brief - Includes a diverse and balanced range of stakeholders - Presents problem analysis - Deliberates on policy options - Uses a facilitator - Not necessarily focusses on consensus | Boydell et al., 2017  Lavis, Boyko, et al., 2009  Moat et al., 2014  Ridde & Dagenais, 2017  van de Kerkhof, 2006 |
| Contribution Mapping | - Moves away from measuring research ‘impacts’ - Uses qualitative research methods to study what concrete contributions are made - Involves extensive fieldwork - Grounded in constructivist understanding of scientific practice | Borst et al., 2019  Kok et al., 2016  Kok & Schuit, 2012 |

**
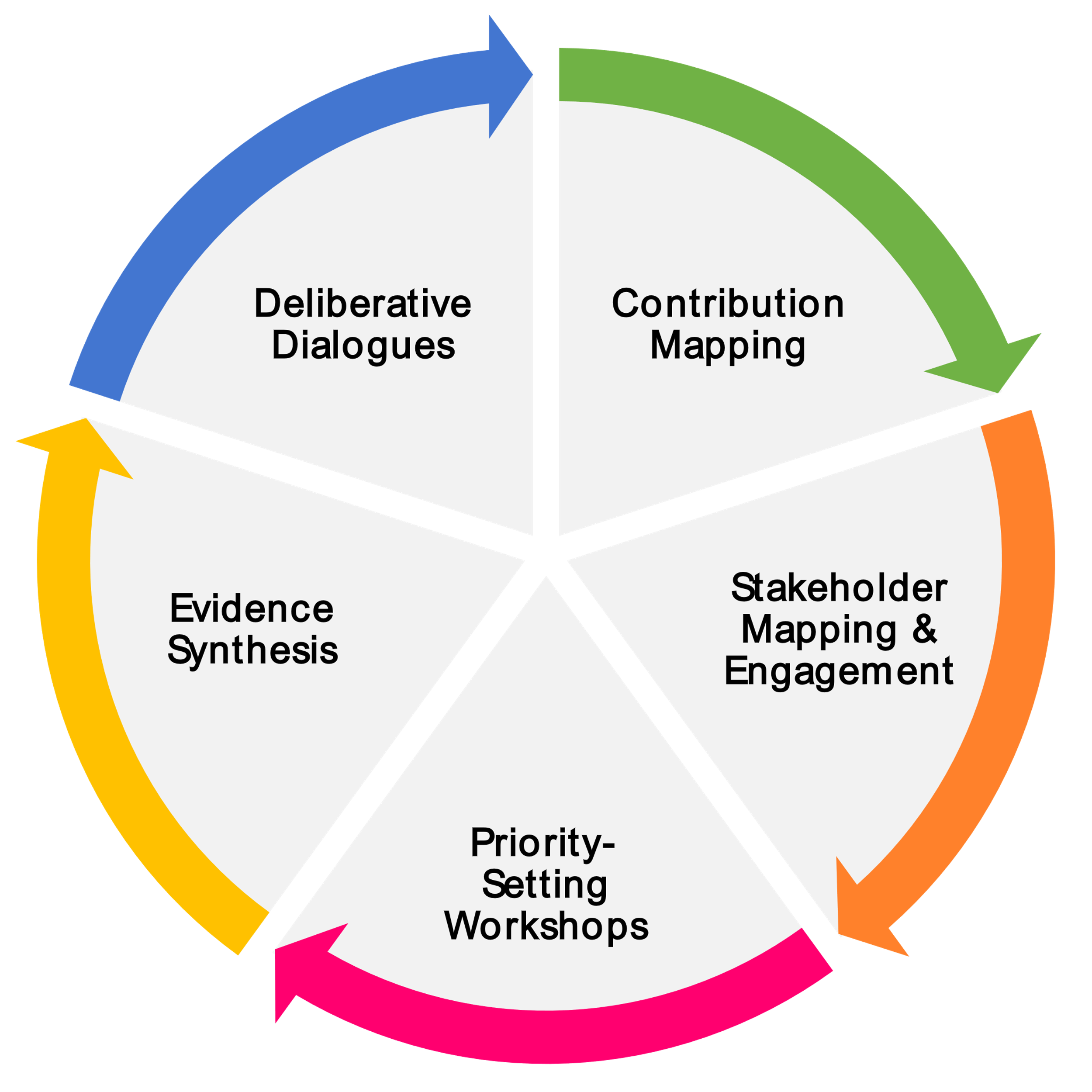
**

**Figure S1. A representation of the cyclical nature of the knowledge translation processes that we organised. Although stakeholder mapping is commonly seen as the starting point, this figure emphasises that the process can start at any point – depending on what work already exists in the environment where the framework is applied.**

**References for further reading**

Armstrong, R., Hall, B. J., Doyle, J., & Waters, E. (2011). ‘Scoping the scope’ of a cochrane review. *Journal of Public Health*, *33*(1), 147–150. https://doi.org/10.1093/pubmed/fdr015

Boaz, A., Hanney, S., Borst, R., O’Shea, A., Kok, M., O’Shea, A., & Kok, M. (2018). How to engage stakeholders in research: Design principles to support improvement. *Health Research Policy and Systems*, *16*(1), 60. https://doi.org/10.1186/s12961-018-0337-6

Borst, R. A. J., Kok, M. O., O’Shea, A. J., Pokhrel, S., Jones, T. H., & Boaz, A. (2019). Envisioning and shaping translation of knowledge into action: A comparative case-study of stakeholder engagement in the development of a European tobacco control tool. *Health Policy*, *123*(10), 917–923. https://doi.org/10.1016/j.healthpol.2019.07.012

Boydell, K. M., Dew, A., Hodgins, M., Bundy, A., Gallego, G., Iljadica, A., Lincoln, M., Pignatiello, A., Teshima, J., & Willis, D. (2017). Deliberative Dialogues Between Policy Makers and Researchers in Canada and Australia. *Journal of Disability Policy Studies*, *28*(1), 13–22. https://doi.org/10.1177/1044207317694840

Kok, M. O., Gyapong, J. O., Wolffers, I., Ofori-Adjei, D., & Ruitenberg, J. (2016). Which health research gets used and why? An empirical analysis of 30 cases. *Health Research Policy and Systems*, *14*(1), 36. https://doi.org/10.1186/s12961-016-0107-2

Kok, M. O., & Schuit, A. J. (2012). Contribution mapping: A method for mapping the contribution of research to enhance its impact. *Health Research Policy and Systems*, *10*(1), 1–16. https://doi.org/10.1186/1478-4505-10-21

Lavis, J. N., Boyko, J. A., Oxman, A. D., Lewin, Simon., & Fretheim, Atle. (2009). SUPPORT Tools for evidence-informed health Policymaking (STP) 14: Organising and using policy dialogues to support evidence-informed policymaking. *Health Research Policy and Systems*, *7*(Suppl 1), 1–8. https://doi.org/10.1186/1478-4505-7-S1-S14

Lavis, J. N., Permanand, G., Oxman, A. D., Lewin, S., & Fretheim, A. (2009). SUPPORT Tools for evidence-informed health Policymaking (STP) 13: Preparing and using policy briefs to support evidence-informed policymaking. *Health Research Policy and Systems*, *7*(Suppl 1), 507–513. https://doi.org/10.1186/1478-4505-7-S1-S13

Levac, D., Colquhoun, H., & O’Brien, K. K. (2010). Scoping studies: Advancing the methodology. *Implementation Science*, *5*(1), 69. https://doi.org/10.1186/1748-5908-5-69

Moat, K. A., Lavis, J. N., Clancy, S. J., El-Jardali, F., Pantoja, T., Moat, K. A., Lavis, J. N., Clancy, S. J., El-Jardali, F., & Pantoja, T. (2014). Evidence briefs and deliberative dialogues: Perceptions and intentions to act on what was learnt. *Bulletin of the World Health Organization*, *92*(1), 20–28. https://doi.org/10.2471/BLT.12.116806

Oliver, K., Kothari, A., & Mays, N. (2019). The dark side of coproduction: Do the costs outweigh the benefits for health research? *Health Research Policy and Systems*, *17*(1), 33. https://doi.org/10.1186/s12961-019-0432-3

Petticrew, M., & Roberts, H. (2006). *Systematic reviews in the social sciences: A practical guide*. Blackwell Pub.

Pratt, B. (2019). Towards inclusive priority-setting for global health research projects: Recommendations for sharing power with communities. *Health Policy and Planning*. https://doi.org/10.1093/heapol/czz041

Ridde, V., & Dagenais, C. (2017). What we have learnt (so far) about deliberative dialogue for evidence-based policymaking in West Africa. *BMJ Global Health*, *2*(4), e000432. https://doi.org/10.1136/bmjgh-2017-000432

Smith, V., Devane, D., Begley, C. M., & Clarke, M. (2011). Methodology in conducting a systematic review of systematic reviews of healthcare interventions. *BMC Medical Research Methodology*, *11*(1), 15. https://doi.org/10.1186/1471-2288-11-15

van de Kerkhof, M. (2006). Making a difference: On the constraints of consensus building and the relevance of deliberation in stakeholder dialogues. *Policy Sciences*, *39*(3), 279–299. https://doi.org/10.1007/s11077-006-9024-5

Viergever, R. F., Olifson, S., Ghaffar, A., & Terry, R. F. (2010). A checklist for health research priority setting: Nine common themes of good practice. *Health Research Policy and Systems*, *8*(1), 36. https://doi.org/10.1186/1478-4505-8-36
